# Molecular diagnosis of Chagas disease in patients with megaesophagus exhibiting negative or inconclusive serological results

**DOI:** 10.3389/fpara.2025.1622149

**Published:** 2025-08-13

**Authors:** Angelica Martins Batista, Tycha Bianca Sabaini Pavan, Eros Antonio de Almeida, Daniel Maximo Corrêa de Alcantara, Paula Durante Andrade, Luiz Cláudio Martins, Jamiro da Silva Wanderley, Sandra Cecília Botelho Costa, Gláucia Elisete Barbosa Marcon

**Affiliations:** ^1^ Center of Biological and Health Sciences, Estácio de Sá University, Rio de Janeiro, RJ, Brazil; ^2^ Advanced Public Health Laboratory, Gonçalo Moniz Institute, Oswaldo Cruz Foundation, Salvador, BA, Brazil; ^3^ Grupo de Estudos em Doença de Chagas (GEDoCh), Department of Medical Clinic, Faculty of Medical Sciences, State University of Campinas, Campinas, São Paulo, SP, Brazil; ^4^ Oswaldo Cruz Foundation Mato Grosso do Sul, Campo Grande, MS, Brazil; ^5^ Laboratory of Diagnosis of Infectious Diseases by Molecular Biology Techniques, Faculty of Medical Sciences, State University of Campinas, Campinas, SP, Brazil

**Keywords:** Chagas megaesophagus, negative serology, inconclusive serology, molecular diagnostic, nPCR, qPCR

## Abstract

Chagasic megaesophagus is a relatively uncommon clinical manifestation in individuals with chronic Chagas disease (CD), and it has not been extensively documented in literature. However, individuals may exhibit negative or inconclusive serology for CD. This study aimed to assess the performance of molecular diagnostics for CD in participants with these conditions. This was a prospective cohort study that included 26 participants with negative or inconclusive conventional CD serology (Group I), 33 participants with positive CD serology and megaesophagus (Group II), and 10 participants with negative serology and no CD epidemiological history (Group III). Blood samples were collected for serological tests (ELISA and IFAT), blood cultures, and molecular tests like nested PCR (nPCR) targeting Sat-DNA and kDNA, as well as quantitative PCR (qPCR) of *T. cruzi*. Statistical analyses applying the Composite Reference Standard (CRS), showed that diagnosis by Sat-DNA nPCR had a sensitivity of 95% (95% CI: 82%–99%), a specificity of 81% (95% CI: 64%–93%), an accuracy of 88%. When considering a positive result from at least one molecular test, 20 out of 26 participants with megaesophagus and negative or inconclusive conventional serology were identified (76.9%). This study reinforce the greater detection capacity of Sat-DNA nPCR compared to the diagnostic methods tested. This emphasizes the importance of employing molecular diagnosis to clarify the etiology in megaesophagus cases.

## 1 Introduction

Chagas disease (CD) is caused by the protozoan *Trypanosoma cruzi* (*T. cruzi*), which was first described in 1909 by Carlos Chagas (1909). This disease is classified by the World Health Organization (WHO) as the third most neglected disease worldwide, behind malaria and schistosomiasis (Dias et al., 2016; World Health Organization (WHO), 2023). More than a century after its discovery, CD still affects 5–6 million individuals in several Latin American countries and remains one of the leading causes of sudden death, arrhythmias, and heart failure (Martins-Melo et al., 2014). It continues to be associated with high rates of morbidity and mortality (World Health Organization (WHO), 2023).

CD is an infectious condition with two phases: acute and chronic. In the first few weeks, the disease manifests in its acute form, causing symptoms like fever, fatigue, and malaise, with the potential for severe clinical conditions like meningoencephalitis or myocarditis. However, in most cases, it remains asymptomatic (Dias et al., 2016; World Health Organization (WHO), 2023). In the chronic phase, which can occur decades after infection, individuals may develop complications resulting from direct parasite damage, inflammation, fibrosis of affected organs, or neurovegetative lesions. About 10% of infected individuals experience digestive tract alterations, leading to conditions like megaesophagus or megacolon, which constitute the clinical digestive form of CD (Salvador et al., 2014). In Brazil alone, this group comprises approximately two million individuals. Chronic CD also affects the hearts of 30% of infected individuals (Rassi et al., 2010).

Megaesophagus is the most common manifestation of the clinical digestive form of chronic CD. Its typical symptoms include dysphagia and regurgitation, which are similar to those seen in idiopathic (primary) achalasia and other causes of esophageal dilatation. An accurate etiological diagnosis is required to distinguish between these conditions (Dantas, 2003; Herbella et al., 2004; Borges Migliavaca et al., 2018).

Parasitological, serological, and more recently, molecular tests are employed to establish a CD diagnosis, each method with its respective inherent limitations (Schijman, 2018). Parasitological tests (xenodiagnosis and blood cultures) exhibit high specificity, but limited sensitivity during the chronic phase of CD, and they are not readily available for diagnosis (Britto et al., 1995; Junqueira et al., 1996). Serology is a suitable and accessible method for diagnosing CD, particularly in the chronic phase, but false-negative and false-positive results can occur due to cross-reactions with various trypanosomatids and different *T. cruzi* lineages (Batista et al., 2010; Baldoni et al., 2023). This issue is especially clear in cases of megaesophagus, where clinical and epidemiological markers align with CD, yet serological tests consistently yield negative results, complicating differentiations from other potential causes. In the 1990s, as molecular techniques advanced, polymerase chain reaction (PCR) tests emerged as the preferred method for diagnosing *T. cruzi* infection (Moser et al., 1989), proving to be the most effective option for this purpose.

Although the use of two conventional serological tests based on different principles is currently considered the consensus for laboratory diagnosis of the chronic phase of CD (Ministério da Saúde, 2018), molecular tests such as conventional PCR (Moser et al., 1989; Sturm et al., 1989) and quantitative PCR (qPCR) (Piron et al., 2007; Duffy et al., 2013; Moreira et al., 2013) are possibly relevant as complementary tools in CD diagnosis. This is due to their ability to detect *T. cruzi* DNA, especially in cases of inconclusive serology (Marcon et al., 2002), in immunosuppressed patients (de Freitas et al., 2011), or when monitoring therapeutic failure is desired (Pavan et al., 2023). The sensitivity of PCR in the chronic phase ranges from 40–70%, due to low and intermittent parasitemia, requiring standardization and optimization strategies for the samples (Pascual-Vázquez et al., 2023).

The qPCR testing was employed to determine the etiology of *T. cruzi* in a group of individuals with megaesophagus and negative CD serology. DNA amplification was detected also via nested PCR (nPCR) in 76% of the samples, particularly those with a positive CD epidemiological history (Batista et al., 2010).

Molecular methods have allowed medical professionals to quantify *T. cruzi* DNA using qPCR, constituting an advancement in diagnosing CD in specific situations, depending on the disease stage and available protocols (Piron et al., 2007; Pinazo et al., 2010; Hernández et al., 2016).

Chagasic megaesophagus is primarily associated with the destruction of the intramural nervous plexus of the esophagus, which leads to impaired motility and subsequent dilation of the organ. Epidemiological studies report that this condition affects approximately 2% to 8.8% of individuals with the chronic form of the disease, with higher prevalence observed in endemic regions such as Brazil (Barros et al., 2019). In this context, the use of PCR as a differential diagnostic tool in patients with chagasic megaesophagus emerges as a promising strategy. This molecular approach is particularly valuable in addressing the limitations of conventional serology, which frequently produces negative or inconclusive results in such cases (Batista et al., 2010). The implementation of PCR-based diagnostics contributes significantly to enhancing diagnostic accuracy and supports the timely initiation of appropriate therapeutic interventions.

Given that megaesophagus serves as a clinical marker for CD, this study aimed to evaluate the diagnostic performance of a parasitological methods, such as blood culture (BC) and molecular tests, including nPCR and qPCR in individuals with megaesophagus and nonreactive or inconclusive conventional serological results for *T. cruzi*.

## 2 Materials and methods

### 2.1 Study design

This was a prospective cohort study conducted on patients with megaesophagus who had negative or inconclusive serology for CD. The study was supervised by the Study Group on Chagas Disease (GEDoCh) at the University of Campinas (UNICAMP) in Brazil between 2009 and 2013. Information on the clinical form and epidemiological data of the participants were obtained from the medical records of the Clinical Hospital of UNICAMP. After receiving detailed explanations, participants provided their informed consent by signing a consent form. The study received approval from the Research Ethics Committee of UNICAMP (process no. 779/2007). All procedures adhered to the guidelines and standards for research involving human subjects, as outlined in Resolution No. 466/2012 of the Brazilian National Health Council and the principles of the Declaration of Helsinki, ensuring the protection of participants’ rights and well-being.

In this study, blood culture (BC) and molecular diagnostic techniques, including nested PCR (nPCR) and quantitative PCR (qPCR), were applied to patients with megaesophagus who presented negative or inconclusive serological results for Chagas disease. The samples were obtained from patients enrolled in a prospective study and attending a specialized outpatient clinic. Due to limitations in the volume of blood that could be collected, a maximum of 20 mL was allocated for BC. Additionally, 4 mL of blood was collected in tubes with a clot activator and sent to the Clinical Pathology Laboratory of the Clinical Hospital at UNICAMP for analysis using indirect immunofluorescence (IFAT) and enzyme-linked immunosorbent assay (ELISA). Another 4 mL was collected in EDTA tubes for genomic DNA extraction, yielding a final elution volume of 50 μL. This volume was used to perform the following molecular assays: PCR targeting the β-globin gene, followed by nPCR for the Sat-DNA target, nPCR for the kDNA target, and qPCR. However, due to the limited amount of material available, it was not possible to apply all diagnostic methods to every sample, as detailed in the methodological section.

### 2.2 Sampling

**Group I (experimental group):** comprised 26 adult participants of both sexes, diagnosed with megaesophagus and with negative or inconclusive conventional serological for CD;
**Group II (control group):** comprised 33 adult participants of both sexes, diagnosed with megaesophagus and with positive conventional serological for CD.
**Group III (negative control):** comprised 10 adult participants of both sexes, with negative epidemiology and conventional serological for CD, without any gastrointestinal manifestation.

### 2.3 Samples collection

Peripheral blood samples were collected simultaneously for serological, blood culture, and molecular testing. For the serological tests, 4 mL of peripheral blood was drawn into vacuum tubes with a clot activator, which were then processed at the Clinical Pathology Laboratory of the Clinical Hospital at UNICAMP. The enzyme-linked immunosorbent assay (ELISA) and indirect immunofluorescence (IFAT) methods were conducted following the manufacturer’s instructions.

The BC was not performed on 14 patients in group I and on 11 patients in group II due to insufficient blood collection. A total of five vacuum tubes, each containing 4 mL of blood with EDTA, were collected, resulting in 20 mL of peripheral blood. These tubes were then centrifuged at 3,500 rpm for 10 minutes at 4°C. Subsequently, the plasma was discarded, and the remaining red blood cells and coating were washed in a liver infusion tryptose (LIT) culture medium, and then transferred to five culture tubes with 10 mL of LIT in each tube, which were maintained in an incubator at 28°C. Optical microscopy was used for making the evaluations, and this was performed once every two weeks, until positive results were obtained, or until after the 150-day incubation period had passed (Luz et al., 1994).

Molecular testing addressed the performance of qualitative nPCR, targeting the Sat-DNA and kDNA of *T. cruzi*, as well as qPCR. 4.0 mL of peripheral blood was collected in a vacuum tube containing EDTA. For the genetic material isolation, the blood sample was centrifuged at 3,500 rpm for 15 minutes at 4°C, allowing us to extract the buffy coating. The High Pure PCR Template Preparation kit (Roche, Mannheim, Germany) was then used, following the manufacturer’s instructions. After extraction, the genetic material (50µL) was stored at −20°C until it was used to perform the molecular reactions.

### 2.4 DNA amplification

The integrity and absence of inhibitors in the extracted genetic material were confirmed by amplifying the human β-globin gene (Saiki et al., 1985). For β-globin amplification, 1 uL of DNA in a final volume of 20 uL was used for each sample. For this amplification of the Sat-DNA target by nPCR, a mixture of 1.0 μL of DNA, 50 mM of KCl, 10 mM of Tris-HCl (pH 8.4), 2.5 mM of MgCl_2_, 1.5 mM of dNTPs, 0.1 mM of each TCZ1/TCZ2 oligonucleotide, and 2 U of Taq DNA polymerase was prepared in microcentrifuge tubes, resulting in a final volume of 20 μL. For the second reaction, 1.0 μL of the previously amplified product was used as a template, with adjustments to the concentration of MgCl_2_ (2.8 mM) and using TCZ3/TCZ4 oligonucleotides. The amplicon length was 149 bp (**Table 1**). The amplification cycles differed for the two reactions. Both began with an initial denaturation at 94°C for 5 min, followed by a final extension at 72°C for 7 min. In the first reaction, the first 5 cycles were performed at 94°C for 1 min, 60°C for 1 min, and 72°C for 1 min and 30 s. The subsequent 25 cycles were performed at 94°C for 1 min, 65°C for 1 min, and 72°C for 1 min and 30 s. In the second reaction, 25 cycles were performed under the following conditions: 94°C for 40 s; 55°C for 40 s; and 72°C for 1 min (Moser et al., 1989; Ochs et al., 1996; Marcon et al., 2002).

**Table 1:** Nucleotide sequences for nPCR (Sat-DNA and kDNA), and qPCR.

Gene	Denomination	Nucleotide sequence	Reference
Sat-DNA	TCZ1 TCZ2 TCZ3 TCZ4	5’CGAGCTCTTGCCCACACGGGTGCT3’ 5’CCTCCAAGCAGCGGATAGTTCAGG3’ 5’TGCTGCA(G/C)TCGGCTGATCGTTTTCGA3’ 5’CA(A/G)G(C/G)TTGTTTGGTGTCCAGTGTTGTGA3’	(Moser et al., 1989; Ochs et al., 1996; Marcon et al., 2002)
kDNA	S67 S35 S36	5’TGGTTTTGGGAGGGG(C/G)(G/C)(T/G)TCAA(A/C)TTTT3’ 5’AGTACGTAGAG(T/G)GGGCATGTAATAAA3’ 5’GGGTTCGATTGGGGTTGGTGT3’	(Sturm et al., 1989)
qPCR	Cruzi 1 (forward) Cruzi 2 (reverse) Cruzi 3 (probe)	5′-A(C/G)TCGGCTGATCGTTTTCGA-3 5′-AATTCCTCCAAGCAGCGGATA-3 ′ 5′-CACACACTGGACACCAA-3 ‘	(Piron et al., 2007; Duffy et al., 2013)

Sat-DNA, Satellite DNA; kDNA, kinetoplast DNA; qPCR, quantitative PCR.

For the kDNA nPCR, two patients from group I (G1P10 and G1P21) and 6 patients from group II (G2P9, G2P15, G2P16, G2P17, G2P30, G2P33) were not tested due to insufficient samples. To amplify the kDNA target, a combination of 1.0 μL of DNA, 50 mM of KCl, 10 mM of Tris-HCl (pH 8.4), 4 mM of MgCl_2_, 2 mM of dNTPs, 0.1 mM of each S67/S35 oligonucleotide, and 2 U of Taq DNA polymerase was prepared in the microcentrifuge tubes, resulting in a final volume of 20 μL. In the second reaction, 1.0 μL of the previously amplified product was used, along with S35/S36 oligonucleotides (**Table 1**). The amplicon length was 330 bp. The amplification cycles were distinct for the two reactions. Both started with an initial denaturation at 94°C for 5 min, followed by a final extension at 72°C for 7 min. In the first reaction, 35 cycles were employed: 94°C for 1 min; 56°C for 1 min; and 72°C for 1 min. In the second reaction, 35 cycles were conducted under the following conditions: denaturation: 94°C for 30 s; 63°C for 45 s; and 72°C for 1 min (Sturm et al., 1989).

In the nPCRs, the negative control consisted of genetic material derived from a clinical sample with two negative serological tests for Chagas disease and no epidemiological evidence suggestive of the infection. This control was used to verify the absence of reagent or environmental contamination; no amplification was observed in any of the reactions. The positive control contained genetic material from *T. cruzi*, obtained from clinical samples with a reactive serological diagnosis, clinical manifestations compatible with cardiac and/or digestive involvement, and a positive epidemiological history. This ensured that the reagents and reaction conditions were appropriate, with amplification observed at the expected cycles in all reactions. Finally, the no-template control, composed of all nPCR reagents except DNA, was used to confirm the absence of nonspecific amplification and cross-contamination.

Of the 33 samples from group II, four were not tested by qPCR due to insufficient samples (G2P5, G2P16, G2P18, G2P27). For the qPCR test, the data points of the standard curve were derived by counting trypomastigote forms of *T. cruzi* originating from BC in a Neubauer chamber, with the concentration adjusted to 10^7^ parasites/mL. A blood sample from an individual without CD was spiked with the known concentration, and from this aliquot, nucleic acids isolation was performed using the High Pure PCR Template Preparation kit (Roche, Mannheim, Germany). Subsequently, serial dilutions (10X) of the genetic material were prepared at concentrations ranging from 10^6^ to 10^−2^ parasites/mL. These diluted points were used in triplicate to establish the standard curve for qPCR.

For the amplification reaction, we used a TaqMan Universal Master Mixer with UNG (Applied Biosystems, Dubai) at a concentration of 1X. Additionally, 500 nM of each oligonucleotide (Cruzi 1/Cruzi 2), 200 nM of the probe (Cruzi 3) (**Table 1**), and RNaseP (Applied Biosystems) at 0.1X were added to a microcentrifuge tube. To reach a final volume of 50 μl, 5 µL of DNA was included. All points used for constructing the standard curve (ranging from 10^6^ to 10^−2^ parasites/mL) were subjected to amplification in triplicate, and DNA from clinical samples underwent amplification in duplicate (Piron et al., 2007; Duffy et al., 2013; Moreira et al., 2013).

The qPCR test was conducted using a Rotor-Gene 6000 machine (Corbett Life Science, California, USA) under the following conditions: an initial cycle of 2 min at 50°C, followed by a second cycle of 10 min at 95°C, and then cycling 45 times with 15 s at 95°C and 60 s at 58°C. To prevent carryover contamination, the TaqMan^®^ Universal Master Mixer kit, containing AmpErase^®^ UNG, was employed. The recombinant UNG enzyme effectively degrades preamplified DNA fragments, preventing reamplification and potential false-positive results. The human RNaseP gene served as an internal control for the amplification reaction. The absence of contaminants in the reagents was confirmed using a No-Template Control (NTC) sample, which did not contain the target sequence. qPCR data were generated using Rotor-Gene 1.7.87 software. Each reaction included patient samples in duplicate, an intermediate point of the standard curve, and a negative control that showed no amplification, thereby ensuring no contamination.

### 2.5 Statistics

Statistical comparisons between Group I and Group II for all applied tests were performed using either the chi-square test or Fisher’s exact test, as appropriate based on sample size and expected frequencies. Sensitivity and specificity analyses were performed using the Composite Reference Standard (CRS) as the reference, applying the majority rule criterion. Emphasis was placed on the Positive Likelihood Ratio (PLR) to assess the ability of each test to confirm the diagnosis of CD. A PLR greater than 5.0 suggests that Sat-DNA nPCR has moderately strong confirmatory power, representing the best balance between sensitivity and specificity among the methods evaluated. We adopted the majority rule to define the CRS, considering a sample as “positive” when two or more different tests yielded positive results. This approach enables a more robust evaluation in contexts where no true gold standard exists, as is the case with CD. A significance level of α = 0.05 was adopted for all statistical tests. All analyses were performed using R software version 4.4.3 (R Core Team, 2025), with the packages ‘rstatix’ (Kassambara, 2023) for statistical testing and ‘irr’ (Gamer et al., 2019) for inter-rater reliability assessments.

## 3 Results

### 3.1 Conventional serology

Serological tests, specifically the ELISA and IFAT tests for CD, were conducted on all study participants. These tests were carried out by the Clinical Pathology Laboratory at the Clinical Hospital of UNICAMP. The results confirmed negative or inconclusive results for individuals in Group I, positive results for those in Group II, and negative results for Group III.

### 3.2 Blood cultures

Within Group I (n=26), BC tests were conducted on 12 patients, and all of them yielded negative results. In Group II (n=33), BC tests were performed on 22 patients, resulting in positive findings for five cases (22.7%), and negative findings for 17 cases (77.3%). There was no statistical significance between Groups I and II regarding the results of the BC (p-value=0.137) (**Table 2**).

**Table 2:** Blood culture results for Groups I and II.

Group	Positive	Negative	Means days positivity
I	0/12 (0%)	12/12 (100%)	0
II	5/22 (22,7%)	17/22 (77,3%)	58

Blood culture (BC) p-value = 0.137.

### 3.3 Qualitative molecular tests - nPCR

In both Groups I and II, β-globin PCR yielded positive results for all cases. kDNA nPCR was performed in 24 patients in group I and in 27 patients in group II. The positivity rate for kDNA was 34,6% in Group I, and 37% in Group II. The Sat-DNA nPCR was performed in all groups: I (n=26) and II (n=33). The positivity rate for Sat-DNA nPCR was 76.9% in Group I, and 63.6% in Group II. There was no statistical significance observed between the kDNA nPCR results for Groups I and II (p-value=1), which was also true of the Sat-DNA nPCR results (p-value=0.394). When comparing the nPCR results for kDNA and Sat-DNA in Group I and II, we observed a higher positivity rate for the Sat-DNA target relative to kDNA, with a statistically significant difference (p-value=0.0014) (**Table 3**). The Group III, composed of people with negative epidemiology and serology for CD, presented negative results in all PCRs directed at *T. cruzi* targets.

**Table 3:** Conventional and nPCR results for Groups I and II.

Group	β-globin	kDNA			Sat-DNA		
	Positive	Positive	Negative	NR	Positive	Negative	NR
I	26 (100%)	9 (34,6%)	15 (57,6%)	2	20 (76.9%)	6 (23.1%)	0
II	33 (100%)	10 (37%)	17 (63%)	6	21 (63.6%)	12 (36.4%)	0

kDNA, kinetoplast DNA; Sat-DNA, Satellite DNA; NR, Not realized.

### 3.4 Quantitative molecular tests - qPCR

**Figure 1:**
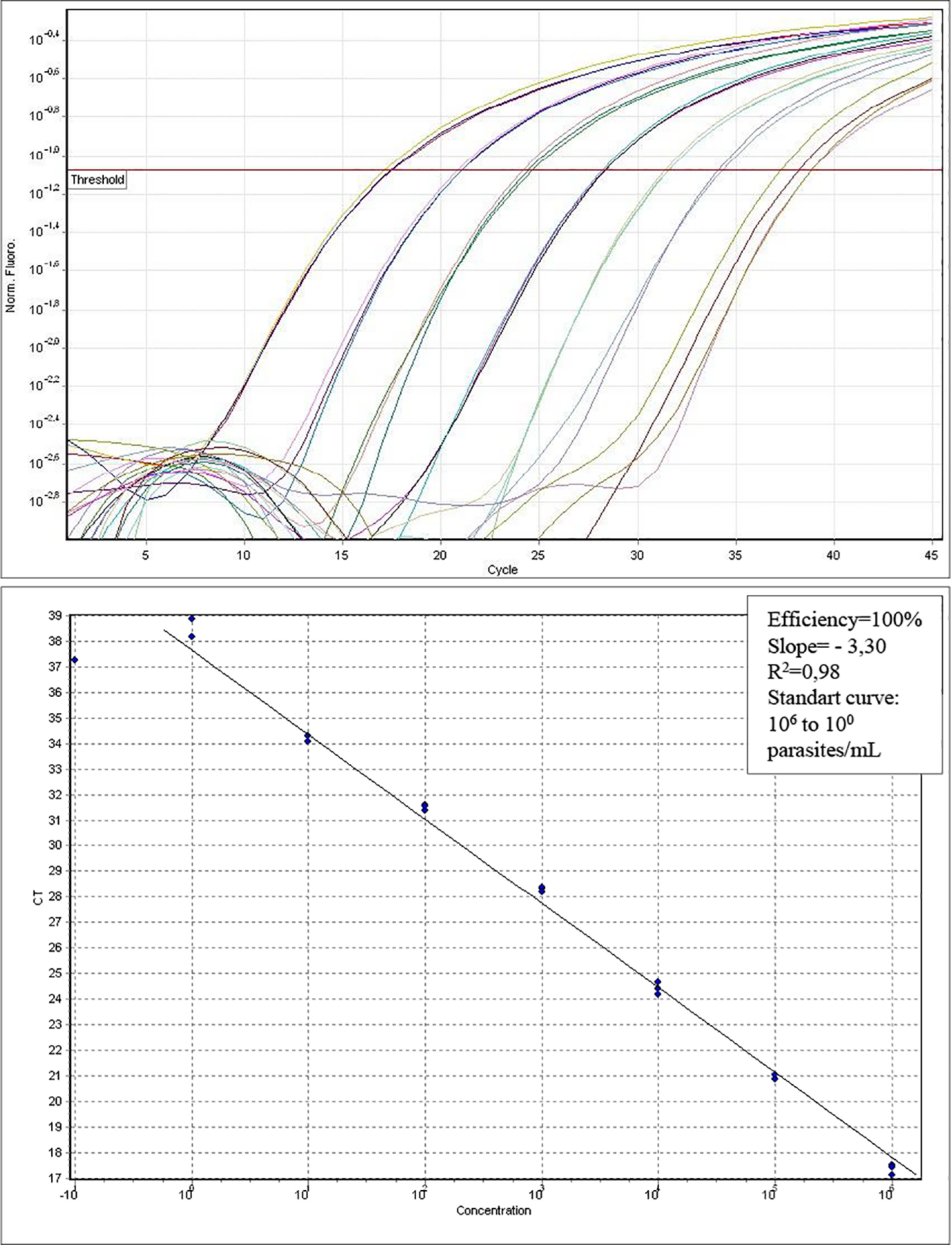
Standard curve for absolute quantification in parasites/ml. Efficiency (E=100%); Slope= -3.30, and R2=0.98. Linearity between seven dilution points in standard curve.

The standard amplification curve displays parameters that are suitable for result reliability. The efficiency was 100% (an ideal value falls between 95 and 100%), the slope was -3,30 (matching the ideal value of -3.32), and R^2^ was 0.98 (an ideal value being 0.99). The linearity observed between points 10^6^ and 10^0^ parasites/mL resulted in a standard curve comprising seven points (**Figure 1**). In the qPCR analysis, samples with values above 0.01 Parasites Equivalents per milliliters (Par.Eq./mL) were categorized as quantifiable. Samples with values equal to or less than 0.01 Par.Eq./mL were considered inconclusive, given the non-quantifiable nature of the parasite load. Samples lacking an amplification signal were classified as being not detectable or negative.

In group I, qPCR detected and quantified 10/26 (38.4%) of the samples, 11/26 (42.3%) were inconclusive, and 5/26 (19.2%) were negative or not detectable (19.2%). In group II, qPCR detected and quantified 6/29 samples (20.7%), inconclusive samples were 10/29 (34.5%), and negative or not detectable samples were 13/29 (44.8%). Considering the detectable, undetectable, and inconclusive results of Groups I and II, there was no statistical difference between the groups (p-value=0.108) (**Table 4**).

**Table 4:** qPCR results for Groups I and II.

Group	Detectable	Inconclusive	Not detectable
I	10 (38.4%)	11 (42.3%)	5 (19.2%)
II	6 (20.7%)	10 (34.5%)	13 (44.8%)

CT, Cycle Threshold; *p-value=0.108.

The sensitivity and specificity analyses of the molecular techniques considered groups I and II. Upon applying the Composite Reference Standard (CRS) with the majority rule criterion, the Sat-DNA nPCR technique demonstrated the best overall performance, with the following estimates: sensitivity of 95% (95% CI: 82%–99%), specificity of 81% (95% CI: 64%–93%), Accuracy rate of 88%, and a PLR of 5.05 (95% CI: 2.44–10.42). Other tests, such as kDNA PCR and qPCR, showed infinite PLR values due to their 100% specificity and absence of false positives. However, their sensitivities were lower (61% and 64%, respectively), which may limit their standalone clinical applicability, particularly in settings where diagnostic exclusion is important. Finally, serological testing showed a reasonable sensitivity (79%) but limited specificity (63%), with a PLR of 2.14, indicating a weak confirmatory capacity (**Table 5**).

**Table 5:** Sensitivity and specificity of the different tests based on a composite reference standard.

Test	Sensitivity (%) [95% CI]	Specificity (%) [95% CI]	Accuracy (%)	PLR [95% CI]
Sat-DNA	95 [82–99]	81 [64–93]	88	5.05 [2.44–10.42]
kDNA	61 [42–78]	100 [88–100]	80	∞
qPCR	64 [43–82]	100 [82–100]	80	∞
Serology	79 [60–92]	63 [42–81]	71	2.14 [1.27–3.62]
Blood culture	21 [7–42]	100 [83–100]	57	∞

Sat-DNA, Satellite DNA; kDNA, kinetoplast DNA; qPCR, quantitative PCR; CI, confidence interval; PLR, Positive Likelihood Ratio. A PLR >10 is considered strong, between 5–10 moderate, and between 2–5 weak. Symbol ∞: absence of false positives.

When considering at least one of the molecular tests, either the qualitative kDNA nPCR or Sat-DNA nPCR tests, or the quantitative qPCR test, the positivity rate in Group I was 76.9% (20/26), and 69.7% (23/33) in Group II. None of the molecular or serological tests yielded positive results in Group III, which comprised individuals who had tested negative for CD (**Table 6**).

**Table 6:** Results of the serological tests (ELISA and IFAT), BC, kDNA nPCR and Sat-DNA nPCR, and qPCR.

Participants	Serological tests	BC	kDNA nPCR	Sat-DNA nPCR	qPCR (Par. Eq./mL) *
G1P1	inconclusive	negative	negative	negative	Inconclusive (0,0031)
G1P2	inconclusive	NR	negative	positive	71,82
G1P3	inconclusive	negative	positive	positive	0,755
G1P4	negative	NR	positive	positive	0,1067
G1P5	negative	NR	negative	negative	Inconclusive (0,01)
G1P6	negative	negative	positive	positive	0,0419
G1P7	negative	NR	positive	positive	Inconclusive (0,002)
G1P8	inconclusive	NR	negative	positive	0,9673
G1P9	negative	NR	negative	positive	Inconclusive (0,005)
G1P10	inconclusive	NR	NR	positive	0,0162
G1P11	negative	NR	positive	positive	Not detectable
G1P12	inconclusive	negative	positive	positive	Inconclusive (0,001)
G1P13	inconclusive	negative	positive	positive	0,0384
G1P14	negative	negative	negative	positive	Inconclusive (0,001)
G1P15	negative	negative	positive	positive	0,0421
G1P16	negative	NR	negative	negative	Not detectable
G1P17	inconclusive	negative	negative	negative	Not detectable
G1P18	inconclusive	negative	negative	positive	Inconclusive (0,0004)
G1P19	inconclusive	negative	negative	positive	Inconclusive (0,001)
G1P20	negative	negative	positive	positive	Inconclusive (0,007)
G1P21	inconclusive	NR	NR	positive	0,4091
G1P22	negative	NR	negative	positive	Inconclusive (0,001)7
G1P23	inconclusive	negative	negative	positive	16,903
G1P24	negative	NR	negative	negative	Not detectable
G1P25	negative	NR	negative	negative	Not detectable
G1P26	inconclusive	NR	negative	positive	Inconclusive (0,001)
G2P1	positive	NR	negative	negative	Not detectable
G2P2	positive	negative	positive	positive	Inconclusive (0,0002)
G2P3	positive	NR	negative	negative	1,15
G2P4	positive	negative	negative	negative	Inconclusive (0,007)
G2P5	positive	negative	negative	negative	NR
G2P6	positive	NR	negative	positive	Inconclusive (0,0001)
G2P7	positive	negative	negative	negative	Not detectable
G2P8	positive	negative	positive	positive	Not detectable
G2P9	positive	NR	NR	negative	Inconclusive (0,001)
G2P10	positive	positive	positive	positive	2,14
G2P11	positive	negative	negative	positive	Inconclusive (0,002)
G2P12	positive	NR	negative	positive	Not detectable
G2P13	positive	positive	negative	positive	Not detectable
G2P14	positive	positive	positive	positive	0,122
G2P15	positive	NR	NR	negative	Not detectable
G2P16	positive	negative	NR	positive	NR
G2P17	positive	NR	NR	positive	Not detectable
G2P18	positive	negative	negative	negative	NR
G2P19	positive	negative	negative	positive	Inconclusive (0,001)
G2P20	positive	positive	positive	positive	Inconclusive (0,001)
G2P21	positive	positive	negative	positive	Inconclusive (0,0003)
G2P22	positive	negative	negative	negative	Not detectable
G2P23	positive	negative	positive	positive	769,212
G2P24	positive	NR	positive	positive	0,5687
G2P25	positive	negative	positive	positive	0,05687
G2P26	positive	negative	positive	negative	Inconclusive (0,006)
G2P27	positive	NR	negative	negative	NR
G2P28	positive	negative	negative	positive	Not detectable
G2P29	positive	negative	negative	positive	Not detectable
G2P30	positive	negative	NR	positive	Not detectable
G2P31	positive	NR	negative	negative	Not detectable
G2P32	positive	negative	positive	positive	Inconclusive (0,006)
G2P33	positive	NR	NR	positive	Not detectable
G3P1	negative	NR	negative	negative	Not detectable
G3P2	negative	NR	negative	negative	Not detectable
G3P3	negative	NR	negative	negative	Not detectable
G3P4	negative	NR	negative	negative	Not detectable
G3P5	negative	NR	negative	negative	Not detectable
G3P6	negative	NR	negative	negative	Not detectable
G3P7	negative	NR	negative	negative	Not detectable
G3P8	negative	NR	negative	negative	Not detectable
G3P9	negative	NR	negative	negative	Not detectable
G3P10	negative	NR	negative	negative	Not detectable

BC, blood culture; nPCR, nested polymerase chain reaction; kDNA, kinetoplast DNA; Sat-DNA, Satellite DNA; qPCR, quantitative PCR; Par. Eq./mL, Parasites Equivalents per milliliters; G, group; P, participant; NR, not realized. * Par. Eq./mL > 0.01 – quantifiable; Par. Eq./mL ≤ 0.01 inconclusive. Not detectable – without signal of the amplification.

## 4 Discussion

The digestive form of CD often goes unnoticed within the complex framework of American trypanosomiasis. This is primarily because it is less common and takes many years to develop into a severe condition. The participants in this study had megaesophagus as a clinical marker, which was confirmed via radiological examinations. Most of them exhibited positive epidemiological indicators for CD, but their conventional serological tests for trypanosomiasis were either nonreactive or inconclusive. This unique condition sets them apart from other megaesophagus causes, particularly idiopathic achalasia.

Parasitological tests, like xenodiagnosis and BC, could potentially aid in diagnosing CD. However, these are not entirely practical given to their limited availability, low sensitivity, complex execution procedures, and delayed results. In this study, BC were used to make comparisons with molecular tests. This yielded positive results for only five cases (21.73%) for individuals in Group II who had confirmed cases of the digestive form of Chagas disease. However, BC did not provide any clarity regarding Chagas disease for all cases of megaesophagus with negative or inconclusive CD serology in Group I, thereby prolonging diagnostic uncertainty. Literature has consistently documented the low sensitivity of BC testing, possibly due to the low parasitemia in chronic CD, intermittent parasitism (Castro and Prata, 2000), and differing DTUs (Zingales, 2018). The diagnosis of Chagas disease by either hemoculture or molecular methods depends on the presence of parasites in the bloodstream, which circulate intermittently in individuals with chronic Chagas disease. Moreover, parasite circulation is influenced by the life cycle of the parasite and the immunological balance between parasite and host. Polymerase chain reaction (PCR) requires a small blood volume, offers a shorter processing time, and presents a lower risk of contamination, in addition to having higher sensitivity compared to hemoculture. In contrast, hemoculture relies on optimal conditions for parasite growth, requires a larger sample volume, and involves a longer time for parasite detection and analysis (D’Ávila et al., 2018; Nielebock et al., 2021).

The advent of molecular biology techniques in the 1990s has led to significant progress in diagnosing parasitic and infectious diseases. To date, qPCR has been used not only to confirm cases of CD reactivation, but also to monitor trypanocidal treatment efficacy, assess cures or therapeutic failures, or to simply diagnose CD. It is efficient, sensitive, and reproducible, especially when detecting low parasitic loads. It can even reveal equivalent levels of *T. cruzi* DNA below 1 parasite/ml (Sturm et al., 1989; Marcon et al., 2002; Piron et al., 2007; Duffy et al., 2013; Moreira et al., 2013).

The DNA samples underwent both qualitative tests and absolute quantification in real-time. Of the 26 samples quantified in Group I, only two (samples G1P2 and G1P23) showed quantifications above 1.0 Par.Eq./mL of blood. This low parasitemia aligns with the probability of this group comprising chronic patients with non-reactive or inconclusive serology for Chagas disease. Interestingly, in this study, the average parasite load in Group II was 2.45 Par.Eq./mL. Previous research, chronic patients with Chagas heart disease who are seropositive for Chagas disease exhibited an average parasite count of 0.1 Par.Eq./mL. In a previous study involving 50 Brazilian patients, *T. cruzi* Sat-DNA was detected in 31 individuals (62%) using SYBR Green-based qPCR assays (Moreira et al., 2013). In the present investigation, which employed the TaqMan methodology and focused on chronic patients seropositive for the digestive form of Chagas disease, qPCR detected *T. cruzi* DNA in 6 out of 29 patients, corresponding to a positivity rate of 20.7%. Among Group I patients, who presented negative or inconclusive serology, 10 out of 26 (38.4%) showed quantifiable results. It is important to note that the clinical profiles of the evaluated patients differed, potentially influencing diagnostic outcomes due to intrinsic factors such as genetic background and parasitemia levels (Castro and Prata, 2000; Zingales, 2018). Furthermore, the SYBR Green qPCR system is generally considered less specific than the TaqMan-based qPCR approach (Duffy et al., 2013), which may also contribute to variations in sensitivity and detection rates between studies.

The nPCR, using the Sat-DNA or kDNA targets, proved to be suitable for diagnostic purposes in both Groups I and II, with no statistical significance. However, the 95% sensitivity (95% CI: 82%–99%) of the Sat-DNA nPCR indicates a high ability to detect true positive cases, which is crucial for minimizing false negatives, particularly in clinical settings. Although the specificity was moderate (81%; 95% CI: 64%–93%), the Positive Likelihood Ratio (PLR = 5.05; 95% CI: 2.44–10.42) reflects a moderately strong confirmatory capacity.

A meta-analysis revealed that the prevalence of gastrointestinal manifestations in individuals with CD is 12%, with megaesophagus and megacolon being the main clinical forms presented. Among individuals with the digestive form, only 10% receive a diagnosis and only 1% are treated (Baldoni et al., 2023). Due to its complexity and clinical diversity, certain aspects of CD, such as megaesophagus with non-reactive conventional serology for trypanosomiasis, are still not fully understood. *T. cruzi* comprises six genetic lineages, denoted as DTUs I to VI (Zingales, 2018). Additionally, *T. cruzi* exhibits genetic variability among its lineages. One plausible hypothesis for explaining negative serology in cases of megaesophagus, is the potential association of DTUs V and VI with the digestive form of CD (Monje-Rumi et al., 2020). Some serological tests may fail to identify antibodies produced in response to epitopes unrecognized by conventional serology. Recent studies have explored the use of immunodominant epitopes among DTUs to enhance specificity in serological tests, thereby advancing the serological diagnosis of CD (Bhattacharyya et al., 2018; Elisei et al., 2018).

The epidemiological profile of chagasic megaesophagus in central Brazil has been previously outlined. However, individuals with megaesophagus and negative Chagas disease (CD) serology were not included (de Souza et al., 2013). Study conducted by our group, demonstrated positive PCR results in patients with megaesophagus despite negative CD serology (Batista et al., 2010). Reports exist of the progression of chagasic megaesophagus in individuals with negative serology, indicating that 7% of those with chagasic esophagopathy had negative serological results (Castro et al., 1987; Castro et al., 1994). In this study, serological testing demonstrated a reasonable sensitivity (79%) but low specificity (63%), resulting in PLR of only 2.14. This value reflects a weak confirmatory capacity and limits the utility of serology as a standalone diagnostic tool. These findings corroborate the results of this study, which employed molecular biology tools to diagnose Chagas infection in a cohort of patients with megaesophagus and negative or uncertain CD serology, with the majority having positive epidemiological indicators.

In this study, the in-house qPCR assay demonstrated low positivity in quantifying *T. cruzi* DNA extracted from whole blood samples of patients with megaesophagus and inconclusive or negative serology for Chagas disease. Within this group, 42.3% of the results were inconclusive. Among patients with positive serology and megaesophagus, 34.4% also presented inconclusive qPCR results. These findings may be attributed to the low parasitemia typically observed in the chronic phase of Chagas disease, the presence of different *T. cruzi* discrete typing units (DTUs), and technical factors such as sample volume, nucleic acid extraction efficiency, and equipment sensitivity (Schijman, 2018; Schijman et al., 2022). The increased sensitivity of nPCR is likely due to the internal reamplification of the target sequence. Thus, the use of nPCR represents a viable alternative for resolving cases with inconclusive serological results for Chagas disease (Marcon et al., 2002; Dias et al., 2016).

The results in Group II - the digestive form of chronic CD - mirrored the results in Group I, which were characterized by negative or inconclusive serology and megaesophagus. Positivity in at least one of the qualitative or quantitative PCRs enhanced positivity, emphasizing the crucial role of molecular techniques in further examining inconclusive cases of megacolon carriers. This finding reinforces the added value of including molecular methods to achieve greater diagnostic accuracy. Thus, the results obtained using the CRS support the diagnostic superiority of Sat-DNA nPCR in terms of overall performance and clinical applicability.

It is important to point out that improvement in molecular tests, standardizations, and the development of diagnostic kits for commercial use, may all have a positive impact on research and in healthcare services related to CD (Moreira et al., 2023). Confirming the diagnosis of CD in cases of megaesophagus with conventional negative or inconclusive serology can better guide the clinical management of patients, positively impacting their quality of life.

## Data Availability

The original contributions presented in the study are included in the article/supplementary material. Further inquiries can be directed to the corresponding authors.
